# Elucidating Sugar–Acid Metabolic Diversity and Screening Breeding Materials in Xinjiang Pear (*Pyrus*) Germplasm Resources

**DOI:** 10.3390/foods14193354

**Published:** 2025-09-27

**Authors:** Shikui Zhang, Shaopeng Wang, Shangdong Wang, Jinchao Xie, Amanguli Wusiman, Weiquan Zhou

**Affiliations:** 1 National Fruit Tree Germplasm Resources Luntai Fruit Tree Resource Garden, Sciences Fruit and Vegetable Research Institute of Xinjiang Uygur Autonomous Region Academy of Agricultural Sciences, Urumqi 830000, China; wangsp7905@xaas.ac.cn (S.W.); 18139106584@163.com (S.W.); 18999667057@163.com (J.X.); 13629968525@163.com (A.W.); 2 College of Horticulture, Xinjiang Agricultural University/Xinjiang Engineering Research Center for Efficient Cultivation and High-Value Utilization of Forest and Fruit Crops, Urumqi 830000, China

**Keywords:** quality, sensory analysis, fruit ripening period, flavor research

## Abstract

To elucidate the flavor substance basis of the pear germplasm resources in Xinjiang, this study conducted precise qualitative and quantitative analysis of sugars and organic acids in the fruits of 29 pear cultivars. Fructose and glucose are the dominant sugars, accounting for 64.0% of the total sugar content. Malic acid is the dominant organic acid, accounting for 85.8% of the total acid content. The cultivar LL exhibited the highest total sugar content at 633.6 mg·g^−1^, while cultivar JJL-1 showed the highest total acid content at 1441.3 μg·g^−1^. Early-ripening (ER) cultivars demonstrated significantly higher sucrose content compared to mid-ripening (MR) and late-ripening (LR) cultivars, while late-ripening cultivars contained the highest total acid content. These findings provide essential phenotypic data for understanding the genetic basis of sugar and acid metabolism in pear fruits and establish a scientific foundation for parent selection in breeding high-quality pear cultivars in Xinjiang.

## 1. Introduction

Pears belong to the Rosaceae family and the *Pyrus* genus, representing economically significant fruit trees in temperate regions. These trees possess extensive genetic resources and maintain a rich cultivation history [1]. Xinjiang, serving as a crucial center for pear species diversity and cultivation in China, features unique climatic conditions that have fostered the development of diverse local pear cultivars with distinctive flavors. This diversity provides valuable material for investigating pear genetic resource variation [2]. These resources are instrumental in pear resource evaluation, development, utilization, and quality breeding. The latest statistics show that the total cultivated area has reached 71,200 hectares, with an annual output of 1.6 million tons. Xinjiang pears not only hold an important position domestically but also serve as the core production area for China’s pear exports. The export volume has long accounted for over 50% of the national total, with products sold to over 30 countries and regions around the world, including North America, the European Union, and Central Asia, significantly enhancing the competitiveness of China’s agricultural products in the international market. The pear industry has become a pillar of Xinjiang’s agricultural economy, playing a crucial role in promoting regional economic development, increasing farmers’ income, and supporting rural revitalization [3]. Pear fruit quality encompasses both internal and external characteristics, with external qualities including fruit color, size, and weight [4]. Internal quality, primarily determined by sugar and organic acid content and proportions, serves as a critical determinant of fruit quality. These components significantly influence fruit texture, marketability, and flavor characteristics [5,6]. Carbohydrates and organic acids constitute essential nutrients in fruits and serve as key indicators for evaluating intrinsic quality and fundamental flavor characteristics [7]. As living standards improve, breeders, processors, and consumers have increasingly prioritized fruit flavor, with particular emphasis on optimizing sugar-acid flavor profiles and enhancing overall flavor quality as primary objectives in fruit quality improvement [8].

The composition of sugars and organic acids exhibits significant variation among different fruit types [9]. Fruits can be classified based on their primary sugar accumulation during ripening into categories such as fructose, glucose, sucrose, and sorbitol accumulation types [10]. These sugars contribute differentially to sweetness, with sucrose serving as the reference value (1.0), while sorbitol, glucose, and fructose have relative sweetness values of 0.5, 0.75, and 1.75, respectively [11]. Sugar proportions vary across tree species and cultivars. For instance, apricots typically contain higher glucose levels compared to fructose [12], whereas peaches display the inverse pattern [13]. Similarly, fruits can be categorized based on their predominant organic acids into citric acid-dominant and malic acid-dominant types. Malic acid predominates in cherries [14] and apples [15], while citrus fruits primarily contain citric acid [16]. The organic acid content demonstrates substantial variation across fruit species, cultivars, tissue types, and developmental stages [17]. Research by Yao et al. [18] revealed that most western pear cultivars contain higher levels of citric acid than malic acid, classifying them as citric acid-dominant. Akagić et al. [19] demonstrated that in mature fruits of 10 western pear cultivars, fructose was the predominant sugar, followed by sorbitol and glucose, with sucrose showing the lowest concentration.

As the core factor to determine consumer acceptance and market competitiveness, pear flavor quality is mainly controlled by the accumulation and balance of sugar and acid components [20]. Xinjiang is the center of diversity for pear species in China, possessing a large number of unique local varieties. Its distinctive arid and semi-arid climatic conditions, such as large temperature differences between day and night and long hours of sunlight, are extremely conducive to the accumulation of sugar in fruits and the formation of flavor substances. This significantly promotes the accumulation of sugar and the synthesis of flavor substances in fruits, making Xinjiang pears often have higher sweetness and a more balanced sugar-acid ratio, thus forming flavor characteristics that better meet modern consumption preferences. This characteristic not only enhances its market appeal but also provides ideal materials for analyzing the regulatory mechanisms of environmental and genetic interactions on fruit quality at the physiological and metabolic levels [21]. While research on pear germplasm genetic diversity has progressed significantly, studies on Xinjiang’s pear germplasm resources show notable limitations. First, there is insufficient systematic analysis of sugar and acid characteristics in relation to the region’s unique ecological conditions and cultivar diversity. Second, modern metabolomics technologies, particularly high-precision mass spectrometry, have been underutilized for precise qualitative and quantitative analyses of sugar and acid components, hampering understanding of flavor formation mechanisms. Consequently, systematic exploration and quality evaluation of Xinjiang’s pear germplasm resources remain inadequate, particularly regarding sugar and organic acid accumulation patterns.

Advanced analytical techniques including liquid chromatography-tandem mass spectrometry (LC-MS/MS) and gas chromatography-tandem mass spectrometry (GC-MS) are now extensively employed to analyze and quantify flavor compounds in fruits and foods, providing essential data for quality enhancement and cultivar identification [22]. By leveraging these technologies, it is possible to comprehensively identify the sugar and organic acid components in Xinjiang pear fruits and achieve precise quantification, thereby deeply understanding the material basis for the unique flavor of these fruits. Therefore, through field investigations and data collection, this study examined 25 local Xinjiang pear cultivars and 4 representative commercial cultivars. The present research utilized GC-MS and LC-MS/MS technologies to precisely quantify sugar and organic acid components and their concentrations in mature pears from these cultivars. This investigation aimed at characterizing the sugar and organic acid composition profiles of Xinjiang pear cultivars, establishing a foundation for targeted breeding programs to enhance fruit quality and efficiently identify superior pear germplasm resources in Xinjiang.

## 2. Materials and Methods

### 2.1. Plant Materials

The study examined 29 pear germplasm resources maintained at the Luntai Fruit Germplasm Resources Garden of the Xinjiang Academy of Agricultural Sciences (E 84°13′, N 41°47′). All materials received uniform cultivation and management to ensure research result accuracy and comparability. The experiment was conducted from July to October 2024. The highest temperature during this period was 40 °C, the lowest was 6 °C, the average temperature was approximately 30 °C, and the average rainfall was 7.6 mm. Harvest dates were recorded at fruit maturity, determined by color, hardness, and soluble solids content. The 29 pear cultivars were categorized based on harvest time into early-maturing (before 20 August), medium-maturing (20 August to 20 September), and late-maturing cultivars (after 20 September) (Table 1), and pictures of the mature fruits of the 29 pear cultivars are shown in Supplementary Figure S1. During the mature stage, for each cultivar, five plants are randomly selected in the orchard, and 20 fruits are randomly collected from each plant. These fruits are then mixed together to form a combined sample containing 100 fruits. Subsequently, the combined sample is subjected to measurement and analysis. One portion underwent sensory evaluation and quality index determination, while another portion was designated for sugar and organic acid detection. Samples were rapidly frozen in liquid nitrogen and stored at −80 °C for subsequent analysis.

### 2.2. Sensory Evaluation

For each cultivar of pear during its ripening period, 30 healthy fruits of uniform size, color, and without mechanical damage were randomly selected from each cultivar and immediately subjected to sensory evaluation. following the methodology described by Lozano et al. [23,24]. The sensory evaluation experiment was conducted in three independent biological replicates. The evaluation team consisted of 10 (5 male and 5 female) trained members. Before the experiment began, all evaluators received unified training, which included: familiarizing with and identifying basic taste sensations such as sourness and sweetness; understanding the corresponding relationship between the scoring criteria and flavor categories (sour: 1.0–2.5 points; sweet and sour: 2.6–5.0 points; sweet and sour: 5.1–7.5 points; sweet: 7.6–10.0 points); and conducting preliminary tasting exercises to ensure the consistency of the scoring criteria.

The formal evaluation was conducted in an independent sensory evaluation room. All fruit samples were randomly coded (using three-digit random numbers) and presented to the evaluators in a completely random order to avoid evaluators developing subjective preferences due to knowing the cultivar information. Evaluators classified the fruit flavor attributes first (sour, sweet and sour, sweet), and then gave a specific score within the corresponding category score range. Each evaluator rated all samples. For the final score of each sample, we first excluded the highest and lowest scores (to eliminate the influence of extreme values), and then calculated the average score of the remaining 8 evaluators’ ratings.

### 2.3. Determination of Appearance Quality and Soluble Solid Content

For each pear cultivar, 10 fruits were measured. Fruit firmness determined with a hardness tester (GY-1, China). Fruit longitudinal and transverse diameters were measured using digital vernier calipers (Mitutoyo, Shanghai, China). Individual fruit weight was measured using an electronic balance (MP2001, Shanghai Hengping, Shanghai, China). The soluble solid content (SSC) was determined using an electronic digital sugar meter PAL-1 (Atago, Tokyo, Japan).

### 2.4. Analysis of Sugar Composition and Content

Fruits were selected with uniform color, size, and without pests or mechanical damage. Pear samples were placed in a fine powder under liquid nitrogen freezing conditions. A 20.0 mg sample of powder was weighed and transferred to a centrifuge tube with 500 μL of pre-cooled methanol: isopropanol: water (3:3:2 *v*/*v*/*v*) extraction solvent mixture. The sample was vortexed thoroughly for 3 min and placed in an ice bath for ultrasonic extraction in an ultrasonic cleaner for 30 min. The tube was centrifuged (4 °C) at 12,000 r/min for 3 min. A 50 μL aliquot of supernatant was collected and mixed with 20 μL of 100 μg·mL^−1^ ribitol solution (internal standard). Subsequently, 100 μL of methoxyamine hydrochloride in pyridine (15 mg·mL^−1^) was added and incubated at 37 °C for 2 h. Then, 100 μL of BSTFA was added and incubated at 37 °C for 30 min to complete derivatization. A 50 μL aliquot of the derivatized solution was diluted to 1 mL with n-hexane, transferred to a brown automatic injection bottle, and stored at 4 °C in the dark prior to GC-MS analysis (Brand: Agilent, Santa Clara, CA, USA, model: 8890-5977b) [25]. Supplementary Figure S2 shows the total ion current (TIC) data of the samples. Sugar contents were expressed as mg·g^−1^ dry weight, DW.

The chromatography–mass spectrometry conditions mainly include: held at 160 °C for 1 min, raised to 200 °C at a rate of 6 °C/min, raised to 270 °C at a rate of 10 °C/min, raised to 320 °C at a rate of 20 °C/min, and kept for 5.5 min. The chromatographic column is DB-5MS (30 m × 0.25 mm × 0.25 μm), the column flow rate is 1 mL/min, the temperature of the quadrupole mass detector is set at 150 °C, the ion source temperature is set at 230 °C, and the temperature of the transmission line is set at 280 °C. The mass spectrometry scanning mode is selected as ion detection mode (SIM).

### 2.5. Analysis of Organic Acid Composition and Content

Fruits with uniform color, size, and without pests or mechanical damage were selected and ground to powder under liquid nitrogen freezing conditions. A 0.05 g sample of powder was accurately weighed into a 2 mL centrifuge tube, mixed with 500 μL of ice-cold 70% (*v*/*v*) aqueous methanol, and immediately vortexed at 2500 r/min for 3 min. The tube was centrifuged at 12,000 r/min for 10 min (4 °C), and 300 μL of supernatant was transferred to a new 1.5 mL microcentrifuge tube. The tube was stored at −20 °C for 30 min, then immediately centrifuged at 12,000 r/min for 10 min (4 °C). Following centrifugation, 200 μL of the resultant supernatant was aliquoted into LC-MS vials [26]. Supplementary Figure S3 shows the total ion current (TIC) data of the samples. Organic acid contents were expressed as μg·g^−1^ dry weight, DW.

The liquid phase conditions mainly include (1) Chromatographic column: ACQUITY HSS T3 column (1.8 µm, 100 mm × 2.1 mm i.d.); (2) Mobile phase: A phase, ultra-pure water (0.05% formic acid); B phase, acetonitrile (0.05% formic acid); (3) Gradient elution program: 0 min A/B is 95:5 (*v*/*v*), 8–9.5 min A/B is 5:95 (*v*/*v*), 9.6–12 min A/B is 95:5 (*v*/*v*); (4) Flow rate 0.35 mL/min; Column temperature 40 °C; Injection volume 2 μL. The mass spectrometry conditions mainly include Electrospray Ionization (ESI) temperature 550 °C, mass spectrometry voltage 5500 V in positive ion mode, −4500 V in negative ion mode and curtain gas (CUR) 35 psi. In Q-Trap 6500+, each ion pair is scanned and detected based on the optimized de-clustering potential (DP) and collision energy (CE) for each ion pair.

### 2.6. Data Analysis

All the data are represented by the average value obtained from three repetitions. All three biological replicate data were collated using Microsoft Excel 2022, a one-way ANOVA was conducted using SPSS 23.0 software, and the Duncan test at the 0.05 level was analyzed. Statistical analyses were performed using Origin 2024 software. The results of principal component analysis and correlation were visualized using the Chiplot online platform (https://www.chiplot.online, accessed on 1 April 2025).

## 3. Results

### 3.1. Phenotypic Trait Analysis and Sensory Evaluation of Pear Germplasm Resources

The quality characteristics of different pear cultivars exhibited considerable variation (Table 1). The ripening period of 29 pear cultivars ranged from 27 July to 7 October, with variations in fruit weight, longitudinal diameter, transverse diameter, hardness, and soluble solids. Single fruit weight ranged from 47.2 g to 355.0 g, showing significant differences between cultivars. The soluble solids content ranged from 8.8% (‘JJL-1’, ‘HSL’) to 16.3% (‘DSL’). Fruit hardness ranged from 5.1 to 14.6 kg/cm^2^. These significant differences in quality indices among the tested cultivars were primarily attributed to their diverse genetic origins

### 3.2. Analysis of Sugar Composition and Content Variation

The sugar components in pear germplasm resources were analyzed using GC-MS. Twenty-five sugar components were identified (Supplementary Table S1). Total sugar content varied among different pear cultivars at maturity, ranging from 434.9–633.6 mg·g^−1^ FW, with a mean value of 559.2 mg·g^−1^ (Table 2).

**Figure 1:**
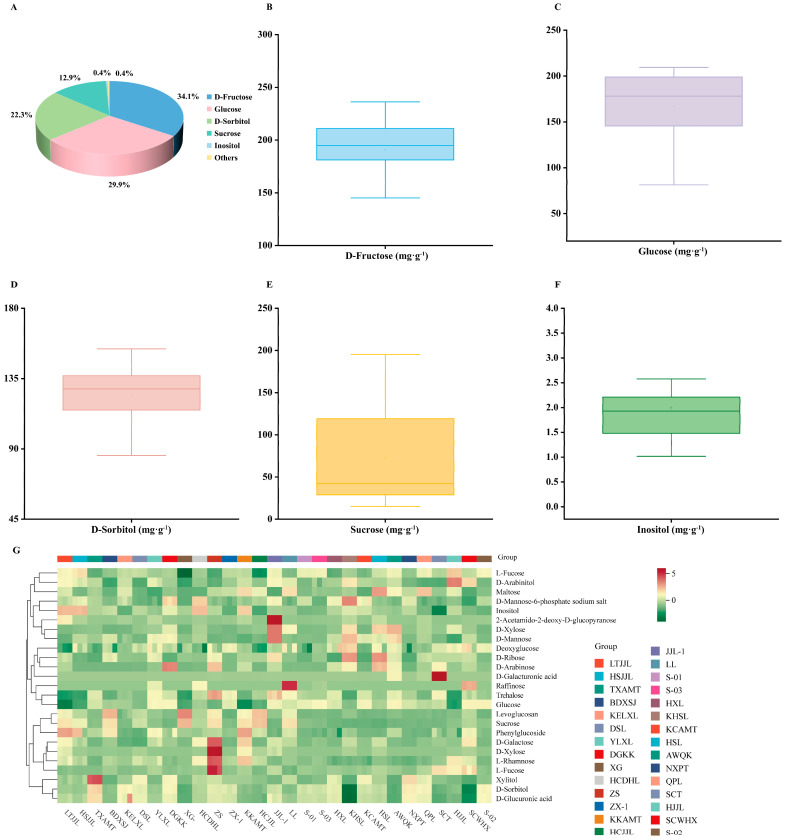
Proportion (**A**), distribution (**B**–**F**) and cluster heat map (**G**) of sugar components in 29 pear fruits. Other components in A represent sugar components excluding glucose, D-fructose, D-sorbitol, sucrose and inositol. The horizontal line in the box indicates the mean value, CV denotes the coefficient of variation, each scatter point represents the content of individual pear cultivars, and the line on the right depicts the fitted distribution curve.

Analysis revealed that the sugar components in the fruits of pear germplasm resources primarily consisted of D-Fructose, glucose, D-Sorbitol, sucrose, and inositol, comprising 34.1%, 29.9%, 22.3%, 12.9%, and 0.4% of the total sugar content, respectively. These five sugars constituted 99.6% of the total, while other components represented only 0.4% (Figure 1A). D-Fructose exhibited the highest content, ranging from 116.0 mg·g^−1^ in ‘XG’ to 236.3 mg·g^−1^ in ‘HJJL’, followed by glucose, ranging from 81.4 mg·g^−1^ (‘LTJJL’) to 209.5 mg·g^−1^ (‘S-02’). Sorbitol content ranged from 75.8 mg·g^−1^ in ‘KHSL’ to 154.0 mg·g^−1^ in ‘TXAMT’, while sucrose content varied from 15.2 mg·g^−1^ FW in ‘HSL’ to 195.2 mg·g^−1^ FW in ‘XG’. Inositol content was comparatively low, ranging from 0.3 mg·g^−1^ in ‘SCT’ to 3.7 mg·g^−1^ in ‘LTJJL’ (Figure 1B–F). The coefficient of variation of D-fructose in the pear germplasm resources was 13.1%, indicating relatively consistent levels of the main sugar components across cultivars.

The cluster heatmap effectively visualizes the subtle variations in sugar components among the fruits of 29 pear germplasm resources. The heatmap was generated based on the components and contents of 25 sugars (Figure 1G). In this visualization, color intensity transitions from green (low content) to red (high content), clearly illustrating the differences in sugar components among the fruits of different pear germplasm resources.

### 3.3. Analysis of the Organic Acids Composition and Content

To characterize the organic acid components and contents of different pear germplasm resources, organic acids were quantified using LC-MS/MS technology to detect the organic acid content in pear fruits. Forty-six organic acid components were identified (Supplementary Table S2), with total acid content ranging from 893.2 to 1441.3 μg·g^−1^, and a mean value of 1136.8 μg·g^−1^ (Table 3).

**Figure 2:**
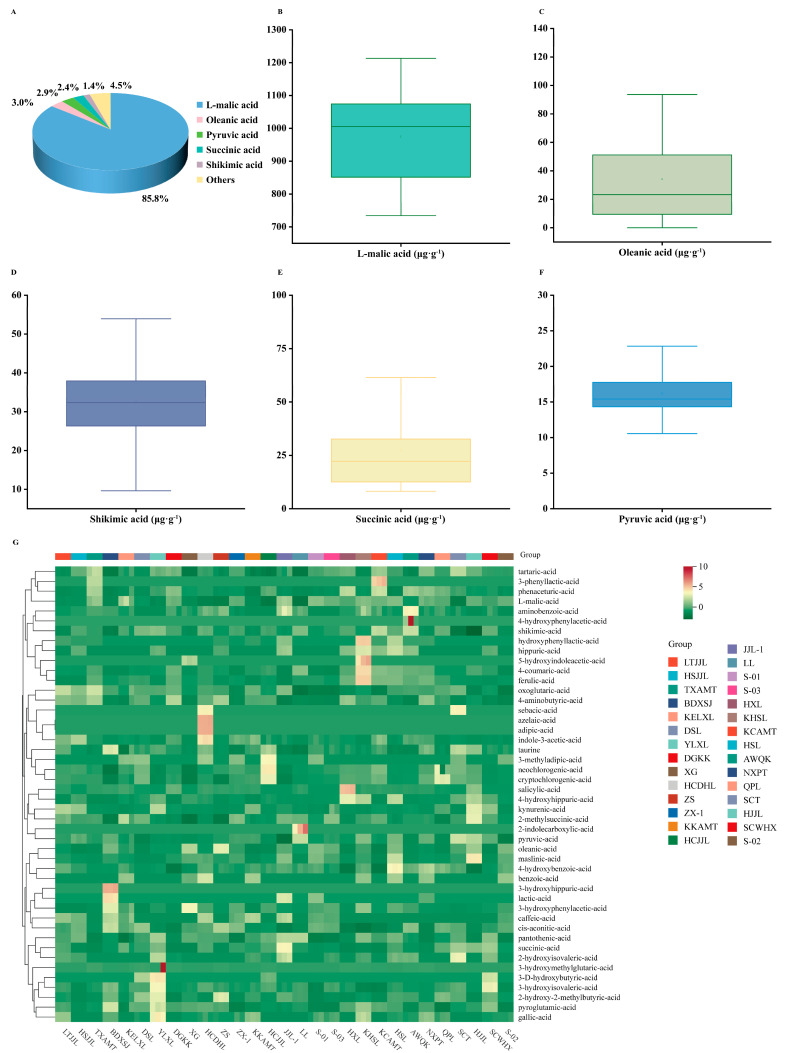
Proportion (**A**), distribution (**B**–**F**) and cluster heat map (**G**) of organic acid components in pear cultivars. The other components in A comprise organic acids excluding L-malic acid, oleanolic acid, shikimic acid, succinic acid and pyruvic acid. The horizontal line in the box indicates the mean value, CV denotes the coefficient of variation, each scatter point represents the content of individual pear cultivars, and the line on the right depicts the fitted distribution curve.

Analysis revealed that the organic acids in pear germplasm resources predominantly comprised L-malic acid, oleanolic acid, succinic acid, pyruvic acid, and shikimic acid, accounting for 85.8%, 3.0%, 2.9%, 2.4%, and 1.4% of the total acid content, respectively. These five organic acids constituted 95.5% of the total, while remaining components represented 4.5% (Figure 2A,G). L-malic acid demonstrated the highest content, averaging 974.9 μg·g^−1^, ranging from 734.5 μg·g^−1^ in ‘LL’ to 1213.2 μg·g^−1^ in ‘JJL-1’. Additionally, trace amounts were detected of oleanolic acid (ranging from undetectable in ‘KCAMT’ to 122.0 μg·g^−1^ in ‘ZS’), succinic acid (8.2 μg·g^−1^ (‘DGKK’)–98.0 μg·g^−1^ (‘JJL-1’)), pyruvic acid (10.6 μg·g^−1^ (‘JJL-1’)–24.3 μg·g^−1^ (‘SCT’)), and shikimic acid (9.6 μg·g^−1^ (‘HJJL’)–55.5 μg·g^−1^ (‘AWQK’)) in the fruits. The mean contents of shikimic acid, oleanolic acid, succinic acid, and pyruvic acid were 32.5 μg·g^−1^, 34.2 μg·g^−1^, 27.4 μg·g^−1^, and 16.2 μg·g^−1^, respectively (Figure 2B–F). Notably, the coefficient of variation of L-malic acid, which constituted the highest proportion of organic acids, was 14.7%, suggesting that organic acids may contribute more significantly to flavor quality than sugars.

The cluster heatmap effectively illustrates the subtle differences in organic acids among the 29 pear germplasm resources fruits. A cluster heatmap was generated based on the components and contents of 46 types of organic acids (Figure 2G).

### 3.4. Variation in Sugar and Organic Acid Contents at Different Maturation Stages

Based on phenological observations, 29 cultivars were categorized into three groups according to their maturity stages (the division criteria are presented in Section 2.1 of the methods): the early-maturing group (ER), comprising 5 cultivars; the medium-maturing group (MR), comprising 11 cultivars; and the late-maturing group (LR), comprising 13 cultivars. To examine variation patterns of organic acid and sugar contents in pear germplasm resources at different maturity stages, comparative analyses were conducted on the sugar content and organic acid content of the three groups.

**Figure 3:**
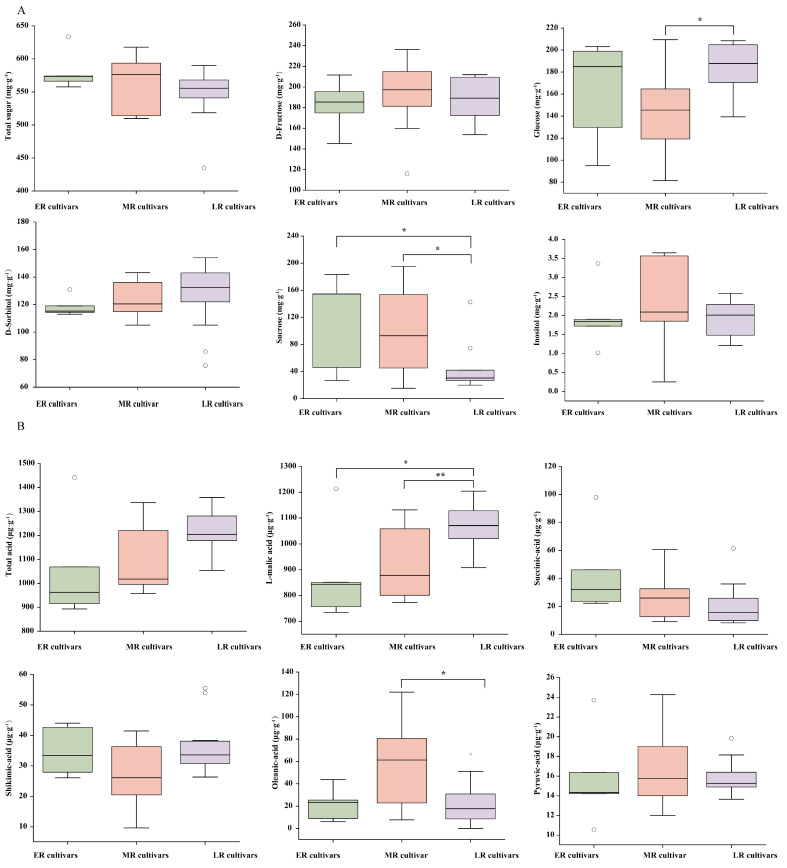
Comparison of sugar and acid component contents in pear fruits across different maturity stages. (**A**) represents sugar components and contents; (**B**) represents organic acid components and contents. The horizontal lines within the boxes indicate median values. The box height equals the interquartile range, representing the distribution of 50% of the data. All cultivars fall within the range, excluding extreme outliers denoted by circles (* *p* < 0.05; ** *p* < 0.01).

Regarding total sugar content, ER cultivars exhibited the highest total sugar content, averaging 581.1 ± 26.9 mg·g^−1^, followed by MR cultivars at 563.9 ± 38.3 mg·g^−1^, while LR cultivars demonstrated the lowest levels at 546.8 ± 37.5 mg·g^−1^. Among individual sugars, the glucose content of the LR cultivars was significantly higher than in MR cultivars (*p* < 0.05), but showed no significant difference from ER cultivars. Additionally, the sucrose content of the ER cultivars was significantly higher than that of both the MR and LR groups (Figure 3A).

Total acid content demonstrated an inverse pattern. LR cultivars displayed the highest total acid content, averaging 1215.2 ± 80.6 μg·g^−1^, followed by MR cultivars at 1080.7 ± 126.1 μg·g^−1^, while ER cultivars showed the lowest levels at 1056.3 ± 201.8 μg·g^−1^. Among individual acids, the L-malic acid content of the LR cultivars was significantly higher than in ER and MR cultivars (*p* < 0.05), and substantially higher than in MR cultivars (*p* < 0.01). Analysis of oleanic acid components revealed that MR cultivars were significantly higher than LR cultivars (Figure 3B).

### 3.5. Correlation Analysis of Fruit Quality Traits in Pear Germplasm Resources

**Figure 4:**
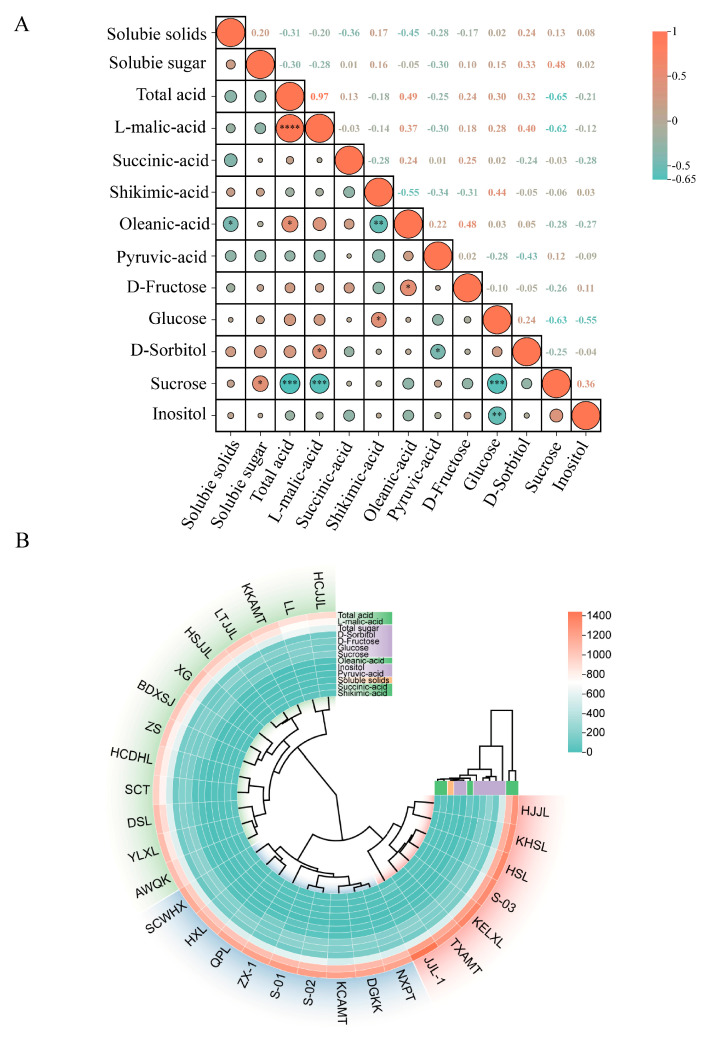
(**A**) Correlation analysis of fruit quality related to pear germplasm resources (* represents a significant difference at *p* < 0.05, ** *p* < 0.01, and *** *p* < 0.001, **** *p* < 0.0001); (**B**) Cluster analysis of pear germplasm resources based on 13 flavor indicators.

As illustrated in Figure 4A, significant correlations exist between the organic acid and sugar components of pear fruit. The total sugar content demonstrated a significant positive correlation with sucrose, while the total acid content showed a highly significant positive correlation with L-malic acid content and a significant positive correlation with oleanic acid content. Shikimic acid content exhibited a significant positive correlation with glucose content. Glucose content showed highly significant positive correlations with inositol and sucrose content, while total acid content demonstrated a highly significant negative correlation with sucrose content. These findings indicate that L-malic acid, oleanic acid, glucose, and sucrose are key components affecting pear fruit quality.

### 3.6. Cluster Analysis

Based on systematic cluster analysis of 13 major flavor indicators, the 29 pear accessions were classified into three groups (Figure 4B). Group I comprised 7 cultivars including ‘HJJL’ and ‘KHSL’, characterized primarily by high acid content, classifying them as a high-acid type. Group II comprised 9 cultivars including ‘NXPT’ and ‘DGKK’, exhibiting relatively high levels of sugar, organic acids, and soluble solids, indicating superior overall flavor quality. Group III included 13 cultivars, such as ‘AWQK’ and ‘YLXL’, characterized by low acid and high sugar content, defining them as a high-sugar type.

### 3.7. Correlation Between Sensory Evaluations and Sugar and Organic Acid Contents

We selected 13 major sugar and acid components and correlated them with the sensory evaluation results through Pearson’s rank correlation analysis, as shown in Supplementary Figure S4. Sour-sweet was significantly positively correlated with succinic acid, D-Fructose, and soluble solids. Sweet-sour was significantly positively correlated with sucrose and oleanic acid. Sour was significantly negatively correlated with succinic acid, total sugar content, and sucrose. Sweet was significantly positively correlated with total sugar content.

## 4. Discussion

Fruit flavor quality is determined by volatile compounds, organic acids, and sugars [27]. Among these components, sugar and organic acids are crucial determinants of fruit quality [28]. Our analysis revealed that malic acid and fructose were the predominant acid and sugar components in 29 pear germplasm resources, consistent with previous studies [29]. This pattern of L-malic acid as the primary organic acid and fructose as the dominant sugar is common in other major horticultural crops such as cherry [14], apple [30], and strawberry [31]. Furthermore, these accumulated sugars and acids themselves are also important precursors of volatile aroma substances such as esters and aldehydes (through fatty acid metabolism and amino acid metabolism pathways), and their content and proportion directly affect the composition of the final aroma profile [32]. The unique ecological conditions in Xinjiang, such as large diurnal temperature differences and abundant sunlight, may have greatly promoted the accumulation and conversion efficiency of these precursor substances, which might be one of the important reasons for the rich flavor of Xinjiang pears [33]. This study enhances our understanding of sugar and organic acid composition in pear fruit from Xinjiang and demonstrates the potential impact of genetic diversity on fruit flavor and quality.

Sugar content and composition are key factors influencing fruit color development, flavor formation, and nutrient accumulation [34]. The sugar composition and content of 29 pear germplasm resources were qualitatively and quantitatively analyzed by GC-MS. Twenty-five sugar components were identified, with total sugar content ranging from 434.9 to 633.6 mg·g^−1^ FW. Fructose and glucose were the predominant sugars, consistent with previous reports [23]. The findings confirm that fructose is the principal sugar in pear fruit. The sugar content of fruits from different cultivars within the same tree species exhibits significant variation, a well-established phenomenon in germplasm resource studies [35]. This variation in sugar content between cultivars is not unique to pear and has been extensively documented in other important horticultural fruit trees, including pomegranate [36] and apple [37]. Notably, sorbitol was detected in all tested pear cultivars. As a common sugar substitute, sorbitol possesses mild laxative and protective properties, and its health benefits warrant greater attention. Lee et al. [38] reported that sorbitol can promote diuresis and aid in reducing blood pressure, benefiting human health. Additionally, Stacewicz-Sapuntzakis reported [39] that diets containing glucose and sorbitol can significantly reduce postprandial blood glucose levels, potentially through sorbitol’s inhibitory effect on glucose production. Therefore, on the basis of a balanced diet, consuming pears in moderation can be regarded as an integral part of a healthy lifestyle.

Organic acids are key components of the pear fruit flavor profile that decisively influence the overall taste of the fruit [40]. In this study, organic acids in 29 pear germplasm resources were qualitatively and quantitatively analyzed using LC-MS/MS. Forty-six organic acids were identified, including L-malic acid, succinic acid, shikimic acid, oleanolic acid, and pyruvic acid. Notably, L-malic acid content predominated, accounting for an average of 85.76%, constituting the primary source of acidity in pear fruit. This finding aligns closely with the analysis results of Li et al. [29] of 81 pear accessions from Sichuan, where malic acid was the most abundant organic acid detected, further confirming malic acid’s role as the core organic acid in pear. However, the total acid content observed in this study ranged from 893.2 μg·g^−1^ (‘LL’) to 1441.3 μg·g^−1^ (‘JJL-1’), significantly lower than the range of 1240~11,920 μg·g^−1^ reported by Wu et al. [26]. This discrepancy may stem from genetic differences among cultivars or environmental factors across cultivation regions.

Key factors influencing fruit flavor and quality include climatic conditions, regional characteristics, and soil environment [41]. The ripening period also influences the flavor and quality of fruits. Sokol-Letowska et al. [42] demonstrated that the ripening period significantly influences total sugar accumulation in sour cherries. Among 21 cultivated cultivars, fruits with medium ripening periods generally exhibited the highest total sugar content. Chen et al. reported that the mean total acid content in four late-maturing pear cultivars was 1.25 times higher than in three mid-maturing cultivars [43]. A comprehensive analysis of organic acids and sugars in the fruits of 29 pear germplasm resources spanning early-maturing (ER), mid-maturing (MR), and late-maturing (LR) periods revealed that total sugar content was highest in early-maturing cultivars (ER), while total acid content was highest in late-maturing cultivars (LR). This finding contrasts with Yin et al., who reported that the total sugar content of late-maturing pear cultivars exceeded that of early-maturing and mid-maturing cultivars [25]. This difference may be attributable to variations in the genetic backgrounds of the studied accessions or the influence of climatic conditions across cultivation regions.

## 5. Conclusions

Through systematic analysis of 29 Xinjiang Pear Germplasm resources, this study revealed significant diversity in sugar and acid metabolism, identifying 25 sugars and 46 organic acids, where the total sugar content ranged from 434.9 to 633.6 mg·g^−1^, with an average value of 559.2 mg·g^−1^. The total acid content ranged from 893.2 to 1441.3 μg·g^−1^, with an average value of 1136.8 μg·g^−1^ and established malic acid and fructose as the dominant components. The analysis determined accumulation patterns characterized by high sugar content in early-maturing cultivars and high acid content in late-maturing cultivars. Cluster analysis enabled the screening of high-sugar (Group III) and high-acid (Group I) resources. These resources are recommended for breeding fresh-market and processing cultivars, respectively. Specifically, high-sugar (Group III) resources are recommended for fresh-market cultivar improvement, while high-acid (Group I) resources are recommended for processing cultivar breeding. This comprehensive analysis and evaluation of pear germplasm resource quality provides valuable reference information for the utilization, breeding, and classification of pear germplasm resources.

## Figures and Tables

**Table 1:** Quality indicators and sensory evaluation of fruits from 29 pear germplasm resources.

Number	Number	Cultivar	Harvest Date	Single-Fruit Weight (g)	Transverse Diameter (mm)	Longitudinal Diameter (mm)	Fruit Shape Index (%)	Fruit Firmness (kg/cm^2^)	Soluble Solids (%)	Sensory Flavor	
										Rating	Score
1	LTJJL	Luntai Jujuli	3 September	102.7 ± 0.3 ^ij^	55.3 ± 1.0 ^kl^	57.8 ± 1.3 ^k^	1.0 ± 0.0 ^cdef^	7.6 ± 0.4 ^ghi^	13.4 ± 0.3 ^cde^	sour–sweet	6.1
2	HSJJL	Hese Jujuli	19 September	109.2 ± 0.7 ^hij^	60.0 ± 0.4 ^ijk^	65.4 ± 0.4 ^ghi^	0.9 ± 0.0 ^defg^	6.9 ± 0.7 ^ij^	12.0 ± 0.4 ^fghi^	sweet–sour	4.8
3	TXAMT	Taxamut	1 October	157.0 ± 0.6 ^defg^	64.7 ± 1.9 ^fghi^	73.0 ± 4.5 ^efgh^	0.9 ± 0.1 ^efgh^	14.6 ± 0.3 ^a^	15.5 ± 0.2 ^ab^	sour	1.6
4	BDXSJ	Baodaoxinshiji	4 September	355.0 ± 7.0 ^a^	85.3 ± 0.8 ^bc^	77.0 ± 2.9 ^cde^	1.1 ± 0.0 ^b^	8.7 ± 0.7 ^g^	12.1 ± 0.6 ^fgh^	sour–sweet	7.1
5	KELXL	Korla Fragrant Pear	23 September	140.7 ± 2.3 ^efghij^	61.0 ± 0.6 ^hij^	73.7 ± 3.8 ^def^	0.8 ± 0.1 ^gh^	5.9 ± 0.3 ^jkl^	12.4 ± 0.4 ^efgh^	sweet	7.1
6	DSL	Dangshanli	18 September	303.2 ± 47.9 ^b^	88.2 ± 0.2 ^b^	90.8 ± 2.8 ^b^	1.0 ± 0.0 ^cde^	12.1 ± 0.4 ^cde^	16.3 ± 0.7 ^a^	sour–sweet	7.0
7	YLXL	Ili Fragrant Pear	14 August	127.5 ± 11.0 ^ghij^	56.7 ± 0.3 ^jkl^	57.5 ± 1.7 ^k^	1.0 ± 0.0 ^c^	12.7 ± 0.6 ^bcd^	13.2 ± 0.6 ^def^	sweet–sour	4.8
8	DGKK	Daguokuikeamut	1 October	151.3 ± 0.8 ^defgh^	45.6 ± 1.1 ^m^	44.8 ± 0.8 ^l^	1.2 ± 0.0 ^b^	12.6 ± 0.4 ^bcd^	14.5 ± 0.2 ^bcd^	sour	2.4
9	XG	Xingao	4 September	189.0 ± 0.6 ^d^	74.0 ± 0.6 ^e^	62.0 ± 0.6 ^ijk^	0.7 ± 0.0 ^i^	8.9 ± 0.1 ^g^	11.9 ± 0.1 ^fghi^	sweet–sour	3.0
10	HCDHL	Huocheng Donghuangli	2 September	191.4 ± 30.2 ^d^	63.3 ± 4.2 ^ghi^	89.3 ± 1.5 ^b^	0.9 ± 0.0 ^efgh^	13.1 ± 0.2 ^bcd^	12.5 ± 0.1 ^efgh^	sweet	8.1
11	ZS	Zaosu	20 September	142.7 ± 3.1 ^efghij^	63.0 ± 1.1 ^ghi^	71.4 ± 2.7 ^defg^	0.9 ± 0.0 ^cdef^	5.1 ± 0.4 ^lm^	11.8 ± 0.2 ^ghi^	sweet–sour	4.8
12	ZX-1	Zhuxuan 1	23 September	190.7 ± 11.7 ^d^	69.7 ± 1.7 ^ef^	74.3 ± 1.5 ^def^	1.0 ± 0.0 ^c^	4.4 ± 0.7 ^m^	14.7 ± 0.7 ^b^	sweet	7.2
13	KKAMT	Kuikeamut	27 July	47.2 ± 0.5 ^k^	45.6 ± 1.1 ^m^	44.8 ± 0.8 ^l^	1.0 ± 0.1 ^c^	12.6 ± 0.4 ^bcd^	14.5 ± 0.2 ^bcd^	sour–sweet	5.8
14	HCJJL	Huocheng Jujuli	2 August	117.7 ± 3.3 ^ghij^	59.3 ± 1.0 ^ijk^	58.7 ± 3.4 ^jk^	1.1 ± 0.1 ^b^	9.0 ± 0.2 ^g^	12.3 ± 0.4 ^efgh^	sweet	7.2
15	JJL-1	Jujuli 1	19 August	101.2 ± 8.9 ^ij^	54.2 ± 0.4 ^l^	49.1 ± 1.7 ^l^	1.1 ± 0.0 ^b^	10.6 ± 0.6 ^f^	8.8 ± 0.1 ^j^	sweet	7.0
16	LL	Lüli	19 August	184.0 ± 17.0 ^de^	73.8 ± 3.5 ^e^	65.1 ± 2.5 ^ghij^	0.9 ± 0.0 ^fgh^	12.1 ± 0.4 ^cdef^	10.7 ± 0.3 ^i^	sweet	6.9
17	S-03	Sha 03	23 September	130.3 ± 2.4 ^fghij^	60.3 ± 0.9 ^hijk^	69.3 ± 0.3 ^fgh^	0.9 ± 0.0 ^efgh^	5.4 ± 0.7 ^klm^	12.2 ± 0.2 ^fghi^	sweet	7.1
18	S-01	Sha 01	23 September	142.7 ± 3.1 ^efghij^	63.0 ± 1.1 ^ghi^	71.4 ± 2.7 ^defg^	0.9 ± 0.0 ^efgh^	5.1 ± 0.6 ^lm^	11.8 ± 0.2 ^ghi^	sweet	8.3
19	HXL	Hongxiangli	7 October	158.7 ± 12.4 ^defg^	62.7 ± 0.9 ^ghi^	70.3 ± 1.5 ^def^	0.9 ± 0.0 ^fgh^	13.4 ± 0.7 ^abc^	14.6 ± 1.1 ^bc^	sour	2.1
20	KHSL	Korla Huangsuanli	23 September	123.0 ± 8.1 ^ghij^	60.3 ± 2.4 ^hijk^	69.7 ± 2.2 ^fgh^	1.0 ± 0.0 ^cdef^	13.9 ± 0.5 ^ab^	9.4 ± 0.6 ^j^	sour	1.9
21	KCAMT	Kuqa Amut	23 September	139.1 ± 4.1 ^efghij^	65.6 ± 1.8 ^fgh^	69.5 ± 2.0 ^fgh^	0.8 ± 0.0 ^gh^	13.4 ± 0.7 ^abc^	11.4 ± 0.3 ^hi^	sour	2.1
22	HSL	Heisuanli	3 September	147.3 ± 3.5 ^defghi^	63.9 ± 1.2 ^ghi^	77.0 ± 0.8 ^cde^	0.8 ± 0.0 ^h^	8.4 ± 0.3 ^gh^	8.8 ± 0.5 ^j^	sour	2.2
23	AWQK	Aiwenqieke	1 October	253.3 ± 27.6 ^c^	78.7 ± 1.2 ^d^	98.0 ± 2.0 ^a^	0.9 ± 0.0 ^defg^	10.8 ± 0.6 ^ef^	16.5 ± 0.2 ^a^	sour–sweet	6.8
24	NXPT	Naxput	1 October	181.7 ± 3.3 ^de^	69.7 ± 2.7 ^ef^	77.0 ± 1.5 ^cde^	1.0 ± 0.0 ^cd^	11.0 ± 0.3 ^ef^	12.8 ± 0.7 ^efgh^	sour	1.9
25	QPL	Qipanli	23 September	157.2 ± 0.8 ^defg^	81.0 ± 0.6 ^cd^	82.0 ± 1.0 ^c^	1.0 ± 0.0 ^cde^	6.8 ± 0.1 ^ijk^	14.3 ± 0.4 ^bcd^	sour–sweet	7.1
26	SCT	Suanchengtuo	20 September	175.0 ± 21.0 ^def^	64.6 ± 2.8 ^fghi^	66.0 ± 2.7 ^ghi^	1.5 ± 0.0 ^a^	10.9 ± 0.9 ^ef^	12.5 ± 0.2 ^efgh^	sour–sweet	6.8
27	HJJL	Huang Jujuli	3 September	97.3 ± 0.7 ^j^	98.3 ± 0.1 ^a^	64.0 ± 1.2 ^hijk^	0.9 ± 0.0 ^efgh^	11.8 ± 0.1 ^def^	11.9 ± 0.1 ^fghi^	sour–sweet	7.0
28	SCWHX	Shache Wanhongxiang	3 October	124.7 ± 0.9 ^ghij^	60.2 ± 0.1 ^hijk^	67.7 ± 0.9 ^fghi^	0.9 ± 0.0 ^fgh^	8.8 ± 0.1 ^g^	12.8 ± 0.1 ^efg^	sour–sweet	7.2
29	S-02	Sha 02	20 September	159.1 ± 3.8 ^def^	67.0 ± 0.9 ^fg^	77.4 ± 0.4 ^cd^	1.0 ± 0.0 ^cdef^	7.2 ± 0.5 ^hij^	13.2 ± 0.2 ^def^	sweet	8.2

Note: Data are expressed as the mean ± standard deviation of 3 biological replicates. Different lowercase letters in the same column indicate significant differences at the 0.05 level, while the same letter’s value represents no statistically significant difference, and data sets with different letters indicate statistically significant differences.

**Table 2:** Sugar components and contents in the fruits of pear germplasm resources.

Cultivar	Total Sugar Content (mg·g^−1^)	D-Fructose (mg·g^−1^)	Proportion (%)	Glucose (mg·g^−1^)	Proportion(%)	D-Sorbitol (mg·g^−1^)	Proportion (%)	Sucrose (mg·g^−1^)	Proportion (%)	Inositol (mg·g^−1^)	Proportion (%)
LTJJL	576.2 ± 1.3 ^e^	214.3 ± 1.2 ^c^	37.2	81.4 ± 1.0 ^t^	14.1	120.4 ± 0.5 ^j^	20.9	153.7 ± 1.3 ^d^	26.7	3.7 ± 0.0 ^a^	0.6
HSJJL	588.9 ± 4.4 ^c^	225.9 ± 2.2 ^b^	38.4	119.2 ± 0.7 ^q^	20.3	118.8 ± 1.0 ^j^	20.2	119.1 ± 0.9 ^f^	20.2	3.6 ± 0.0 ^a^	0.6
TXAMT	549.4 ± 2.2 ^k^	164.8 ± 1.0 ^n^	30.0	184.2 ± 0.8 ^h^	33.5	154.0 ± 1.3 ^a^	28.0	42.2 ± 1.6 ^i^	7.7	2.0 ± 0.0 ^l^	0.4
BDXSJ	617.1 ± 2.8 ^b^	181.2 ± 0.8 ^k^	29.4	162.2 ±1.7 ^l^	26.3	116.1 ± 0.9 ^k^	18.8	153.5 ± 0.9 ^d^	24.9	2.2 ± 0.0 ^g^	0.4
KELXL	541.0 ± 3.4 ^l^	196.3 ± 1.3 ^fg^	36.3	161.8 ±0.3 ^l^	29.9	144.0 ± 1.3 ^c^	26.6	35.6 ± 0.8 ^k^	6.6	1.2 ± 0.0 ^t^	0.2
DSL	576.7 ± 2.8 ^e^	197.3 ± 1.1 ^f^	34.2	145.5 ± 0.8 ^m^	25.2	137.2 ± 0.9 ^e^	23.8	92.8 ± 1.6 ^f^	16.1	2.1 ± 0.0 ^ij^	0.4
YLXL	557.7 ± 0.8 ^ij^	185.4 ± 0.9 ^j^	33.2	203.1 ± 1.6 ^bc^	36.4	119.1 ± 0.5 ^j^	21.4	46.0 ± 1.0 ^h^	8.3	1.7 ± 0.1 ^o^	0.3
DGKK	569.7 ± 2.1 ^fg^	189.1 ± 0.8 ^i^	33.2	205.4 ± 0.9 ^b^	36.1	143.0 ± 1.7 ^cd^	25.1	26.8 ± 0.7 ^mn^	4.7	2.6 ± 0.0 ^d^	0.5
XG	542.0 ± 3.0 ^l^	116.0 ± 0.8 ^q^	21.4	122.1 ± 1.6 ^p^	22.5	105.0 ± 0.8 ^n^	19.4	195.2 ± 0.8 ^a^	36.0	1.9 ± 0.0 ^m^	0.4
HCDHL	553.5 ± 1.8 ^jk^	185.6 ± 0.9 ^j^	33.5	160.7 ± 0.9 ^l^	29.0	124.9 ± 0.5 ^i^	22.6	76.1 ± 0.8 ^g^	13.8	3.6 ± 0.0 ^b^	0.6
ZS	593.5 ± 0.9 ^c^	159.8 ± 0.8 ^o^	26.9	135.4 ± 1.2 ^n^	22.8	136.0 ± 0.8 ^ef^	22.9	156.6 ± 0.8 ^c^	26.4	2.1 ± 0.0 ^jk^	0.4
ZX-1	562.3 ± 0.5 ^hi^	194.9 ± 0.8 ^g^	34.7	191.2 ± 0.8 ^f^	34.0	134.6 ± 1.7 ^f^	23.9	38.4 ± 1.6 ^j^	6.8	1.2 ± 0.0 ^t^	0.2
KKAMT	566.4 ± 3.5 ^gh^	195.4 ± 1.2 ^fg^	34.5	95.0 ± 0.8 ^s^	16.8	115.3 ± 1.2 ^kl^	20.4	154.4 ± 0.8 ^cd^	27.3	3.4 ± 0.0 ^c^	0.6
HCJJL	574.2 ± 3.5 ^ef^	145.1 ± 1.5 ^p^	25.3	129.8 ± 1.8 ^o^	22.6	113.0 ± 1.8 ^l^	19.7	183.2 ± 2.1 ^b^	31.9	1.0 ± 0.0 ^u^	0.2
JJL-1	573.7 ± 1.5 ^ef^	211.5 ± 0.8 ^d^	36.9	198.9 ± 0.4 ^d^	34.7	130.9 ± 1.1 ^g^	22.8	26.7 ± 0.7 ^no^	4.7	1.9 ± 0.0 ^m^	0.3
LL	633.6 ± 0.1 ^a^	174.9 ± 0.9 ^l^	27.6	184.9 ± 0.9 ^h^	29.2	114.4 ± 0.6 ^kl^	18.1	154.7 ± 1.2 ^cd^	24.4	1.8 ± 0.0 ^n^	0.3
S-03	583.0 ± 2.1 ^d^	209.5 ± 0.8 ^d^	35.9	195.7 ± 0.8 ^e^	33.6	132.4 ± 1.7 ^g^	22.7	42.2 ± 0.0 ^i^	7.2	1.4 ± 0.0 ^r^	0.2
S-01	589.9 ± 2.6 ^c^	211.7 ± 1.2 ^d^	35.9	204.8 ± 0.8 ^b^	34.7	131.6 ± 0.8 ^g^	22.3	38.4 ± 1.3 ^j^	6.5	1.5 ± 0.0 ^p^	0.3
HXL	518.5 ± 0.8 ^n^	164.8 ± 1.2 ^n^	31.8	187.8 ± 0.9 ^g^	36.2	136.9 ± 0.9 ^ef^	26.4	24.9 ± 0.7 ^o^	4.8	2.0 ± 0.0 ^k^	0.4
KHSL	434.9 ± 0.9 ^p^	187.4 ± 0.8 ^ij^	43.1	146.7 ± 0.9 ^m^	33.7	75.8 ± 0.8 ^p^	17.4	19.9 ± 0.8 ^p^	4.6	2.5 ± 0.0 ^e^	0.6
KCAMT	529.7 ± 2.7 ^m^	172.5 ± 2.1 ^m^	32.6	201.1 ± 0.8 ^cd^	38.0	131.9 ± 0.9 ^g^	24.9	20.0 ± 0.0 ^p^	3.8	2.3 ± 0.0 ^f^	0.4
HSL	514.0 ± 1.8 ^no^	200.4 ± 0.8 ^e^	39.0	164.7 ± 0.8 ^k^	32.0	128.4 ± 1.2 ^h^	25.0	15.2 ± 0.7 ^q^	3.0	1.9 ± 0.0 ^n^	0.4
AWQK	557.7 ± 4.5 ^ij^	211.9 ± 0.8 ^d^	38.0	208.5 ± 1.7 ^a^	37.4	105.1 ± 1.2 ^n^	18.9	27.4 ± 0.9 ^mn^	4.9	1.7 ± 0.0 ^o^	0.3
NXPT	548.4 ± 0.5 ^k^	181.8 ± 0.7 ^k^	33.2	184.8 ± 0.8 ^h^	33.7	147.1 ± 0.9 ^b^	26.8	30.5 ± 0.8 ^l^	5.6	2.1 ± 0.0 ^i^	0.4
QPL	555.6 ± 1.2 ^j^	211.1 ± 1.2 ^d^	38.0	170.6 ± 1.3 ^j^	30.7	141.2 ± 0.8 ^d^	25.4	28.8 ± 1.3 ^lmn^	5.2	1.4 ± 0.0 ^q^	0.3
SCT	509.8 ± 3.3 ^o^	191.4 ± 0.5 ^g^	37.5	178.2 ± 1.6 ^i^	35.0	108.1 ± 1.2 ^m^	21.2	29.2 ± 0.8 ^lm^	5.7	0.3 ± 0.0 ^v^	0.1
HJJL	513.0 ± 2.3 ^o^	236.3 ± 1.0 ^a^	46.1	112.1 ± 1.7 ^r^	21.9	114.9 ± 1.2 ^kl^	22.4	45.1 ± 0.8 ^h^	8.8	2.2 ± 0.0 ^h^	0.4
SCWHX	568.2 ± 3.3 ^g^	195.6 ± 0.8 ^fg^	34.4	208.3 ± 1.2 ^a^	36.7	85.8 ± 0.8 ^o^	15.1	74.5 ± 0.8 ^g^	13.1	1.5 ± 0.0 ^p^	0.3
S-02	617.8 ± 2.4 ^b^	214.9 ± 0.8 ^c^	34.8	209.5 ± 0.8 ^a^	33.9	143.2 ± 1.6 ^cd^	23.2	47.0 ± 0.8 ^h^	7.6	1.3 ± 0.0 ^s^	0.2
Mean	559.2	190.6	34.2	167.4	30.0	124.5	22.3	75.9	12.7	2.0	0.4
SD	39.0	25.0	5.1	36.7	6.6	17.9	3.1	57.5	9.8	0.8	0.1
CV (%)	7.0	13.1	14.9	21.9	22.0	14.4	13.9	75.8	77.2	40.0	25

Note: Data are expressed as the mean ± standard deviation of 3 biological replicates. Different lowercase letters in the same column indicate significant differences at the 0.05 level, while the same letter’s value represents no statistically significant difference, and data sets with different letters indicate statistically significant differences.

**Table 3:** Organic acid components and contents in the fruits of pear germplasm resources.

Cultivar	Total Acid Content (μg·g^−1^)	L-malic-Acid (μg·g^−1^)	Proportion (%)	Succinic-Acid (μg·g^−1^)	Proportion (%)	Shikimic-Acid (μg·g^−1^)	Proportion (%)	Oleanic-Acid (μg·g^−1^)	Proportion (%)	Pyruvic-Acid (μg·g^−1^)	Proportion (%)
LTJJL	1017.8 ± 7.4 ^jkl^	878.3 ± 7.2 ^gh^	86.3	31.8 ± 1.0 ^fq^	3.1	24.4 ± 0.2 ^o^	2.4	23.2 ± 0.0 ^n^	2.3	15.8 ± 0.1 ^i^	1.6
HSJJL	995.9 ± 1.3 ^kl^	878.0 ± 0.3 ^gh^	88.2	11.8 ± 0.1 ^opq^	1.2	34.8 ± 0.1 ^gh^	3.5	7.7 ± 0.0 ^rs^	0.8	17.8 ± 0.6 ^g^	1.8
TXAMT	1280.7 ± 36.3 ^cde^	1166.1 ± 36.2 ^bc^	91.1	9.6 ± 0.1 ^opq^	0.8	31.3 ± 0.2 ^jk^	2.4	11.5 ± 0.0 ^p^	0.9	15.5 ± 0.2 ^ij^	1.2
BDXSJ	1011.5 ± 5.5 ^jkl^	836.9 ± 6.1 ^hi^	82.7	18.4 ± 0.1 ^kl^	1.8	20.5 ± 0.4 ^p^	2.0	61.3 ± 0.0 ^f^	6.1	17.8 ± 0.0 ^fg^	1.8
KELXL	1358.0 ± 133.7 ^b^	1204.1 ± 127.2 ^ab^	88.7	33.7 ± 11.4 ^ef^	2.5	32.4 ± 3.7 ^ij^	2.4	29.3 ± 4.1 ^l^	2.2	15.3 ± 0.8 ^jk^	1.1
DSL	1054.8 ± 5.5 ^jk^	907.9 ± 5.4 ^g^	86.1	26.0 ± 0.0 ^hi^	2.5	41.5 ± 0.0 ^d^	3.9	9.6 ± 0.0 ^q^	0.9	12.8 ± 0.0 ^q^	1.2
YLXL	1068.9 ± 94.9 ^ij^	843.6 ± 11.1 ^hi^	78.9	46.3 ± 0.5 ^d^	4.3	44.0 ± 0.4 ^c^	4.1	23.3 ± 1.2 ^n^	2.2	14.3 ± 0.1 ^mn^	1.3
DGKK	1197.3 ± 9.2 ^fg^	1074.4 ± 8.9 ^d^	89.7	8.2 ± 0.1 ^q^	0.7	33.6 ± 0.0 ^hi^	2.8	22.0 ± 0.3 ^n^	1.8	14.4 ± 0.1 ^mn^	1.2
XG	1041.8 ± 5.6 ^jk^	857.8 ± 5.0 ^h^	82.3	9.0 ± 0.0 ^pq^	0.9	25.1 ± 0.6 ^no^	2.4	93.7 ± 0.0 ^b^	9.0	14.0 ± 0.0 ^no^	1.4
HCDHL	957.9 ± 1.3 ^lm^	801.3 ± 0.1 ^ij^	83.7	32.7 ± 0.4 ^efg^	3.4	37.9 ± 1.1 ^e^	4.0	22.9 ± 0.4 ^n^	2.4	14.5 ± 0.1 ^m^	1.5
ZS	1006.4 ± 0.3 ^kl^	784.5 ± 0.1 ^j^	78.0	12.6 ± 0.2 ^nop^	1.3	28.2 ± 0.1 ^lm^	2.8	122.0 ± 0.4 ^a^	12.1	15.4 ± 0.0 ^ijk^	1.5
ZX-1	1204.1 ± 6.3 ^fg^	1051.4 ± 6.0 ^de^	87.3	25.9 ± 0.0 ^hi^	2.2	33.9 ± 0.4 ^gh^	2.8	30.9 ± 0.0 ^k^	2.6	16.4 ± 0.1 ^h^	1.4
KKAMT	962.7 ± 1.8 ^lm^	851.5 ± 1.1 ^h^	88.5	23.4 ± 0.1 ^ij^	2.4	26.1 ± 0.7 ^n^	2.7	6.0 ± 0.0 ^t^	0.6	16.4 ± 0.1 ^h^	1.7
HCJJL	915.5 ± 5.7 ^mn^	756.4 ± 4.9 ^jk^	82.6	32.1 ± 0.2 ^fg^	3.5	33.4 ± 0.8 ^hi^	3.7	1.0 ± 0.0 ^qr^	1.0	14.2 ± 0.1 ^mn^	1.6
JJL-1	1441.3 ± 19.5 ^a^	1213.2 ± 21.0 ^a^	84.2	98.0 ± 0.1 ^a^	6.8	28.0 ± 1.0 ^m^	1.9	43.8 ± 0.5 ^h^	3.0	10.6 ± 0.1 ^s^	0.7
LL	893.2 ± 0.1 ^n^	734.5 ± 0.0 ^k^	82.2	22.2 ± 0.1 ^ij^	2.5	42.7 ± 0.0 ^d^	4.8	25.4 ± 0.2 ^m^	2.8	23.7 ± 0.1 ^b^	2.7
S-03	1322.1 ± 4.2 ^bcd^	1149.1 ± 4.0 ^c^	86.9	15.6 ± 0.3 ^lmn^	1.2	28.5 ± 0.0 ^lm^	2.2	66.6 ± 0.0 ^d^	5.0	15.1 ± 0.0 ^kl^	1.1
S-01	1226.4 ± 1.0 ^ef^	1068.5 ± 0.7 ^d^	87.1	17.9 ± 0.1 ^kl^	1.5	30.8 ± 0.0 ^k^	2.5	51.1 ± 0.1 ^g^	4.2	15.7 ± 0.1 ^i^	1.3
HXL	1120.3 ± 0.0 ^hi^	1005.6 ± 0.1 ^f^	89.8	8.7 ± 0.0 ^pq^	0.8	35.1 ± 0.1 ^fg^	3.1	8.1 ± 0.0 ^rs^	0.7	14.9 ± 0.0 ^l^	1.3
KHSL	1296.9 ± 15.6 ^cd^	1127.9 ± 15.4 ^c^	87.0	11.4 ± 0.0 ^opq^	0.9	29.4 ± 0.2 ^l^	2.3	34.4 ± 0.0 ^j^	2.7	19.8 ± 0.0 ^d^	1.5
KCAMT	1212.2 ± 2.8 ^f^	1078.3 ± 1.6 ^d^	89.0	9.9 ± 0.0 ^opq^	0.8	54.0 ± 0.8 ^b^	4.5	0.0 ± 0.0 ^u^	0.0	14.9 ± 0.4 ^l^	1.2
HSL	1336.6 ± 1.1 ^bc^	1131.5 ± 1.0 ^c^	84.7	29.5 ± 0.0 ^gh^	2.2	36.4 ± 0.1 ^e^	2.7	64.6 ± 0.1 ^e^	4.8	19.0 ± 0.0 ^e^	1.4
AWQK	1054.0 ± 0.8 ^jk^	908.2 ± 0.1 ^g^	86.2	16.5 ± 0.1 ^lm^	1.6	55.5 ± 0.2 ^a^	5.3	12.3 ± 0.2 ^p^	1.2	13.7 ± 0.1 ^p^	1.3
NXPT	1198.8 ± 26.1 ^fg^	1071.2 ± 25.5 ^d^	89.4	13.4 ± 0.2 ^mno^	1.1	26.3 ± 0.6 ^n^	2.2	17.7 ± 0.2 ^o^	1.5	18.1 ± 0.7 ^f^	1.5
QPL	1179.1 ± 4.2 ^fg^	1020.9 ± 1.3 ^ef^	86.6	36.0 ± 0.0 ^e^	3.1	38.1 ± 0.3 ^e^	3.2	8.6 ± 0.0 ^qr^	0.7	13.7 ± 0.0 ^op^	1.2
SCT	967.8 ± 0.3 ^lm^	772.8 ± 0.4 ^jk^	79.9	60.7 ± 0.0 ^b^	6.3	17.6 ± 0.0 ^q^	1.8	38.6 ± 0.1 ^i^	4.0	24.3 ± 0.1 ^a^	2.5
HJJL	1276.4 ± 1.2 ^de^	1058.9 ± 1.0 ^de^	83.0	50.9 ± 0.1 ^c^	4.0	9.6 ± 0.0 ^r^	0.8	80.6 ± 0.1 ^c^	6.3	22.8 ± 0.1 ^c^	1.8
SCWHX	1147.4 ± 0.4 ^gh^	979.1 ± 0.2 ^f^	85.3	61.5 ± 0.4 ^b^	5.4	38.3 ± 0.0 ^e^	3.3	7.1 ± 0.0 st	0.6	17.7 ± 0.0 ^g^	1.5
S-02	1220.7 ± 0.6 ^f^	1060.2 ± 0.5 ^de^	86.9	20.5 ± 0.0 ^jk^	1.7	26.1 ± 0.1 ^n^	2.1	61.9 ± 0.0 ^f^	5.1	12.0 ± 0.0 ^r^	1.0
Mean	1136.8	974.9	85.6	27.4	2.4	32.5	2.9	34.2	3.0	16.2	1.5
SD	145.0	142.8	3.3	19.9	1.6	9.5	1.0	29.4	2.7	3.2	0.4
CV (%)	12.8	14.6	3.9	72.6	66.7	29.2	34.5	86.0	90.0	19.8	26.7

Note: Data are expressed as the mean ± standard deviation of 3 biological replicates. Different lowercase letters in the same column indicate significant differences at the 0.05 level, while the same letter’s value represents no statistically significant difference, and data sets with different letters indicate statistically significant differences.

## Data Availability

The original contributions presented in the study are included in the article and Supplementary Materials, further inquiries can be directed to the corresponding author.

## Supplementary Materials

The following supporting information can be downloaded at https://www.mdpi.com/article/10.3390/foods14193354/s1. Supplementary Figure S1. Pictures of the mature fruit of the 29 pear cultivars; Supplementary Figure S2. Shows the sugar total ion current (TIC) data of the samples; Supplementary Figure S3. Shows the organic acid total ion current (TIC) data of the samples; Supplementary Figure S4. Correlation between sensory evaluations and sugar and organic acid contents; Supplementary Table S1. The content of sugar substances detected in the fruits of 29 pear cultivars; Supplementary Table S2. The content of organic acid substances detected in the fruits of 29 pear cultivars.

## References

[1] Potter D., Eriksson T., Evans R.C., Oh S., Smedmark J.E., Morgan D.R., Kerr M., Robertson K.R., Arsenault M., Dickinson T.A. (2007). Phylogeny and classification of Rosaceae. Plant Syst. Evol..

[2] Niu Y.Y., Zhou W.Q., Chen X.Y., Fan G.Q., Zhang S.K., Liao K. (2020). Genome size and chromosome ploidy identification in pear germplasm represented by Asian pears-Local pear varieties. Sci. Hortic..

[3] Liu Y., Wen H., Yang X.P., Wu C.Y., Ming J.Q., Zhang H.Y., Chen J.J., Wang J.B., Xu J. (2023). Metabolome and transcriptome profiling revealed the enhanced synthesis of volatile esters in Korla pear. BMC Plant Biol..

[4] Chen Y.Y., Yin H., Wu X., Shi X.J., Qi K.J., Zhang S.L. (2018). Comparative analysis of the volatile organic compounds in mature fruits of 12 Occidental pear (*Pyrus communis* L.) cultivars. Sci. Hortic..

[5] Huang X.Y., Wang C.K., Zhao Y.W., Sun C.H., Hu D.G. (2021). Mechanisms and regulation of organic acid accumulation in plant vacuoles. Hortic. Res..

[6] He Y.Q., Li Z.Y., Liao G.L., Chen L., Zhong M., Huang C.H., Jia D.F., Xu X.B. (2020). Variation in fruit quality within wild Actinidia eriantha germplasm. N. Z. J. Crop Hortic. Sci..

[7] Zhang Y., Cheng Y., Ma Y., Guan J., Zhang H. (2025). Regulation of pear fruit quality: A review based on chinese pear varieties. Agronomy.

[8] Cao J.P., Jiang Q., Lin J.Y., Li X., Sun C.D., Chen K.S. (2015). Physicochemical characterisation of four cherry species (*Prunus* spp.) grown in China. Food Chem..

[9] Zhou Y., He W.Z., Zheng W.L., Tan Q.L., Xie Z.Z., Zheng C.S., Hu C.X. (2018). Fruit sugar and organic acid were significantly related to fruit Mg of six citrus cultivars. Food Chem..

[10] Famiani F., Bonghi C., Chen Z.H., Drincovich M.F., Farinelli D., Lara M.V., Proietti S., Rosati A., Vizzotto G., Walker R.P. (2020). Stone fruits: Growth and nitrogen and organic acid metabolism in the fruits and seeds—A review. Front. Plant Sci..

[11] Ma Y., Tian T., Zhou J.T., Huang F., Wang Y.Y.K., Liu Y.X., Liu Z.S., He W., Li M.Y., Lin Y.X. (2024). Fruit sugar and organic acid composition and inheritance analysis in an intraspecific cross of Chinese cherry. LWT—Food Sci. Technol..

[12] Xi W.P., Zheng H.W., Zhang Q.Y., Li W.H. (2016). Profiling taste and aroma compound metabolism during apricot fruit development and ripening. Int. J. Mol. Sci..

[13] Zanon L., Falchi R., Santi S., Vizzotto G. (2015). Sucrose transport and phloem unloading in peach fruit: Potential role of two transporters localized in different cell types. Physiol. Plant..

[14] Zhou J.T., Yang S.W., Ma Y., Liu Z.S., Tu H.X., Wang H., Zhang J., Chen Q., He W., Li M.Y. (2023). Soluble sugar and organic acid composition and flavor evaluation of Chinese cherry fruits. Food Chem. X.

[15] Li Y., Yan L., Zhang B., Yang S., Zhao Z. (2021). A study on sugar and organic acid components in different apple cultivars. J. Fruit Sci..

[16] Wang L., Huang Y., Liu Z.A., He J.X., Jiang X.L., He F., Lu Z.H., Yang S.Z., Chen P., Yu H.W. (2021). Somatic variations led to the selection of acidic and acidless orange cultivars. Nat. Plants.

[17] Walker R.P., Battistelli A., Bonghi C., Drincovich M.F., Falchi R., Lara M.V., Moscatello S., Vizzotto G., Famiani F. (2020). Non-structural carbohydrate metabolism in the flesh of stone fruits of the genus *Prunus* (*Rosaceae*)—A review. Front. Plant Sci..

[18] Yao G.F., Yang Z.J., Zhang S.L., Cao Y.F., Liu J., Wu J. (2014). Characteristics of components and contents of organic acid in pear fruits from different cultivated species. Acta Hortic. Sinica..

[19] Akagić A., Oras A., Gaši F., Meland M., Drkenda P., Memić S., Spaho N., Žuljević S.O., Jerković I., Musić O. (2022). A comparative study of ten pear (*Pyrus communis* L.) cultivars in relation to the content of sugars, organic acids, and polyphenol compounds. Foods.

[20] Wang L.B., Ma M., Zhang Y.R., Wu Z.F., Guo L., Luo W.Q., Wang L., Zhang Z., Zhang S.L. (2018). Characterization of the genes involved in malic acid metabolism from pear fruit and their expression profile after postharvest 1-MCP/ethrel treatment. J. Agric. Food Chem..

[21] Li T., Gao Z., Bai X., Yu S., An S., Zheng Q., Tang Z., Zhi J. (2024). Exploring the coupling mode of water and fertilizer for improving growth, fruit quality, and yield of the pear in the arid region. Open Life Sci..

[22] Li W.B., Wu Z.Q., Xu Y., Long H.P., Deng Y.H., Li S.W., Xi Y., Li W.Q., Cai H.L., Zhang B.K. (2023). Emerging LC-MS/MS-based molecular networking strategy facilitates foodomics to assess the function, safety, and quality of foods: Recent trends and future perspectives. Trends Food Sci. Technol..

[23] Lozano L., Iglesias I., Puy J., Echeverria G. (2023). Performance of an expert sensory panel and instrumental measures for assessing eating fruit quality attributes in a pear breeding programme. Foods.

[24] Liu Y., Xiang S.M., Zhang H.P., Zhang H.Y., Wu C.Y., Tang Z.H., Wang J.B., Xu J. (2020). Sensory quality evaluation of korla pear from different orchards and analysis of their primary and volatile metabolites. Molecules.

[25] Yin H., Wu J.Y., Fan J.B., Xu L.L., Zhang W.W., Li Q.H., Jia L.T., Wu X., Wang Z.W., Li H.X. (2024). Profiling of soluble sugar compositions in mature fruits of a diverse pear (*Pyrus* spp.) germplasm by UPLC. J. Food Compos. Anal..

[26] Wu J.Y., Fan J.B., Li Q.H., Jia L.T., Xu L.L., Wu X., Wang Z.W., Li H.X., Qi K.J., Qiao X. (2022). Variation of organic acids in mature fruits of 193 pear (*Pyrus* spp.) cultivars. J. Food Compos. Anal..

[27] Ma W.F., Li B.Y., Zheng L.T., Peng Y.J., Tian R., Yuan Y.Y., Zhu L.C., Su J., Ma F.W., Li M.J. (2021). Combined profiling of transcriptome and DNA methylome reveal genes involved in accumulation of soluble sugars and organic acid in apple fruits. Foods.

[28] Zhang H.P., Wu J.Y., Qin G.H., Yao G.F., Qi K.J., Wang L.F., Zhang S.L. (2014). The role of sucrose-metabolizing enzymes in pear fruit that differ in sucrose accumulation. Acta Physiol. Plant..

[29] Li S.Y., Zhao B., Lu J.Y., Xiang M.J., Lin Y.X., Zhang Y.T., Wang Y., He W., Li M.Y., Chen Q. (2025). Comprehensive analysis and evaluation of the intrinsic fruit quality of Sichuan pear germplasm resources in China. Sci. Hortic..

[30] Ma B.Q., Chen J., Zheng H.Y., Fang T., Ogutu C., Li S.H., Han Y.P., Wu B.H. (2015). Comparative assessment of sugar and malic acid composition in cultivated and wild apples. Food Chem..

[31] Basson C.E., Groenewald J.H., Kossmann J., Cronjé C., Bauer R. (2010). Sugar and acid-related quality attributes and enzyme activities in strawberry fruits: Invertase is the main sucrose hydrolysing enzyme. Food Chem..

[32] Wang X.H., Chen Y.Y., Zhang J.J., Wang Z.W., Qi K.J., Li H.X., Yin H. (2023). Comparative analysis of volatile aromatic compounds from a wide range of pear (*Pyrus* L.) germplasm resources based on HS-SPME with GC–MS. Food Chem..

[33] Liu J.J., Zhang X., Li Z.G., Zhang X.S., Jemric T., Wang X. (2019). Quality monitoring and analysis of Xinjiang ‘Korla’ fragrant pear in cold chain logistics and home storage with multi-sensor technology. Appl. Sci..

[34] Borsani J., Budde C.O., Porrini L., Lauxmann M.A., Lombardo V.A., Murray R., Andreo C.S., Drincovich M.F., Lara M.V. (2009). Carbon metabolism of peach fruit after harvest: Changes in enzymes involved in organic acid and sugar level modifications. J. Exp. Bot..

[35] Tang Y., Ren J., Liu C.X., Jiang J.B., Yang H.H., Li J.F. (2021). Genetic characteristics and QTL analysis of the soluble sugar content in ripe tomato fruits. Sci. Hortic..

[36] Alcaraz-Marmol F., Nuncio-Jauregui N., Garcia-Sanchez F., Martinez-Nicolas J.J., Hernandez F. (2017). Characterization of twenty pomegranate (*Punica granatum* L.) cultivars grown in Spain: Aptitudes for fresh consumption and processing. Sci. Hortic..

[37] Wu J.H., Gao H.Y., Zhao L., Liao X.J., Chen F., Wang Z.F., Hu X.S. (2007). Chemical compositional characterization of some apple cultivars. Food Chem..

[38] Lee J. (2015). Sorbitol, Rubus fruit, and misconception. Food Chem..

[39] Stacewicz-Sapuntzakis M. (2013). Dried plums and their products: Composition and health effects—An updated review. Crit. Rev. Food Sci. Nutr..

[40] Blando F., Oomah B.D. (2019). Sweet and sour cherries: Origin, distribution, nutritional composition and health benefits. Trends Food Sci. Technol..

[41] Yin C., Tian L.M., Li J., Cao Y.F., Dong X.G., Huo H.L., Xu J.Y., Liu C. (2025). Evaluation of pear fruit quality in different ripening stages based on internal quality characteristics. J. Food Compos. Anal..

[42] Sokol-Letowska A., Kucharska A.Z., Hodun G., Golba M. (2020). Chemical composition of 21 cultivars of sour cherry (*Prunus cerasus*) fruit cultivated in Poland. Molecules.

[43] Chen J., Wang Z., Wu J., Wang Q., Hu X. (2007). Chemical compositional characterization of eight pear cultivars grown in China. Food Chem..

