# Supplementary figures and images for: Elucidating Sugar–Acid Metabolic Diversity and Screening Breeding Materials in Xinjiang Pear (Pyrus) Germplasm Resources
Source: Foods. 2025 Sep 27;14(19):3354. doi: 10.3390/foods14193354 (PMC12523242; doi:10.3390/foods14193354)

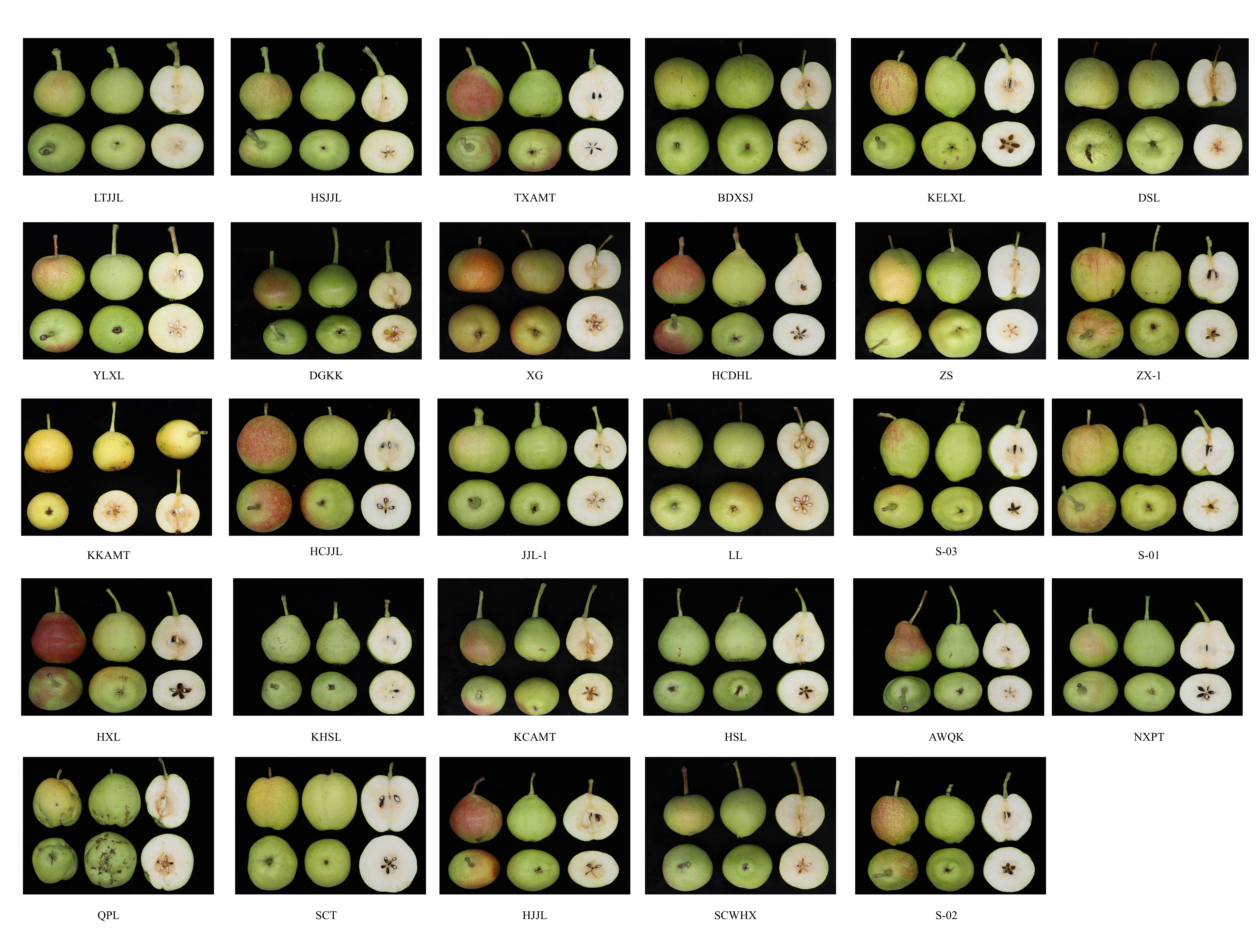

Supplement: Supplementary file 1 [file foods-14-03354-s001.zip › Supplementary Files/Supplementary Figure S1.tif]

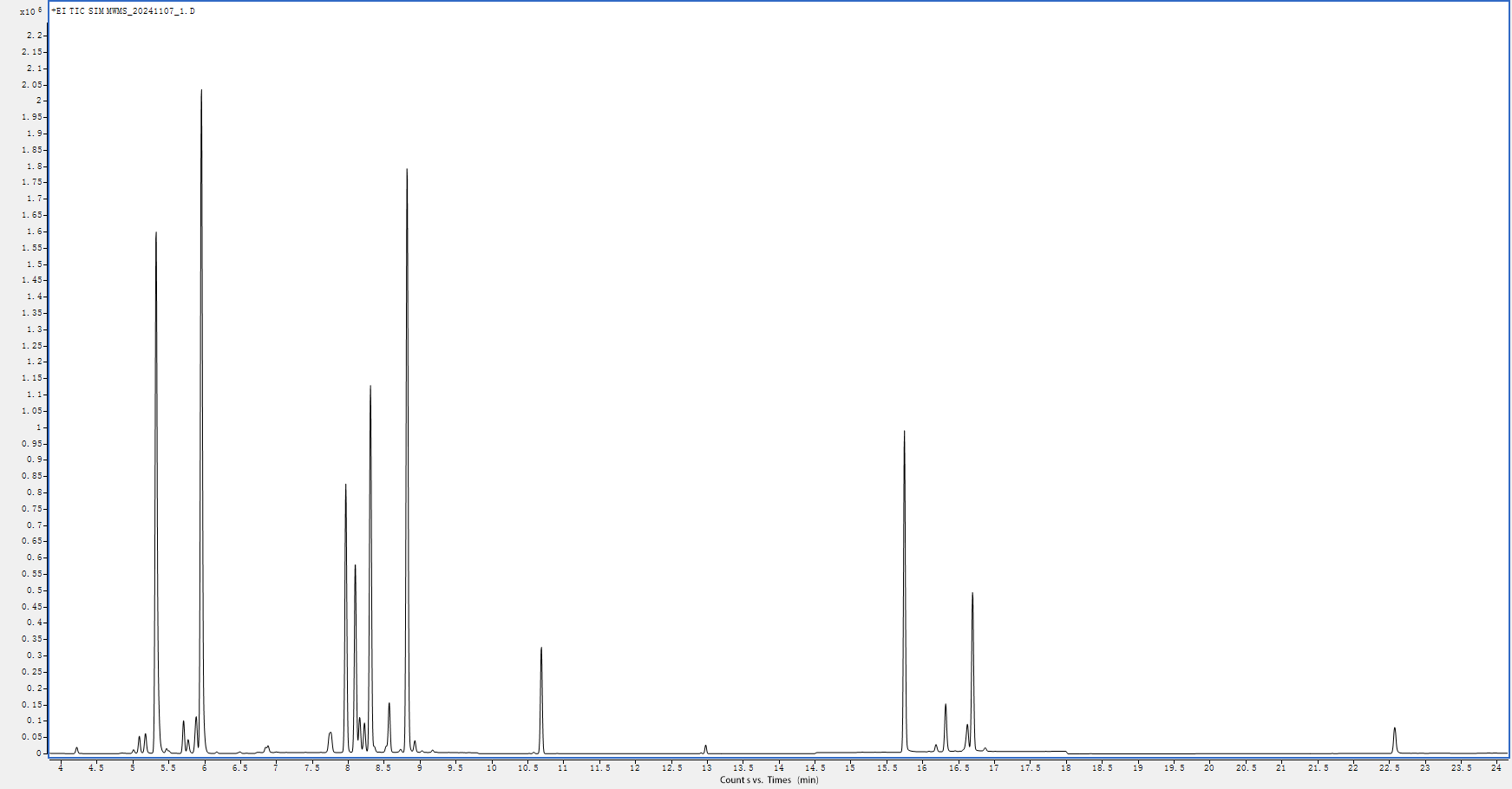

Supplement: Supplementary file 1 [file foods-14-03354-s001.zip › Supplementary Files/Supplementary Figure S2.tif]

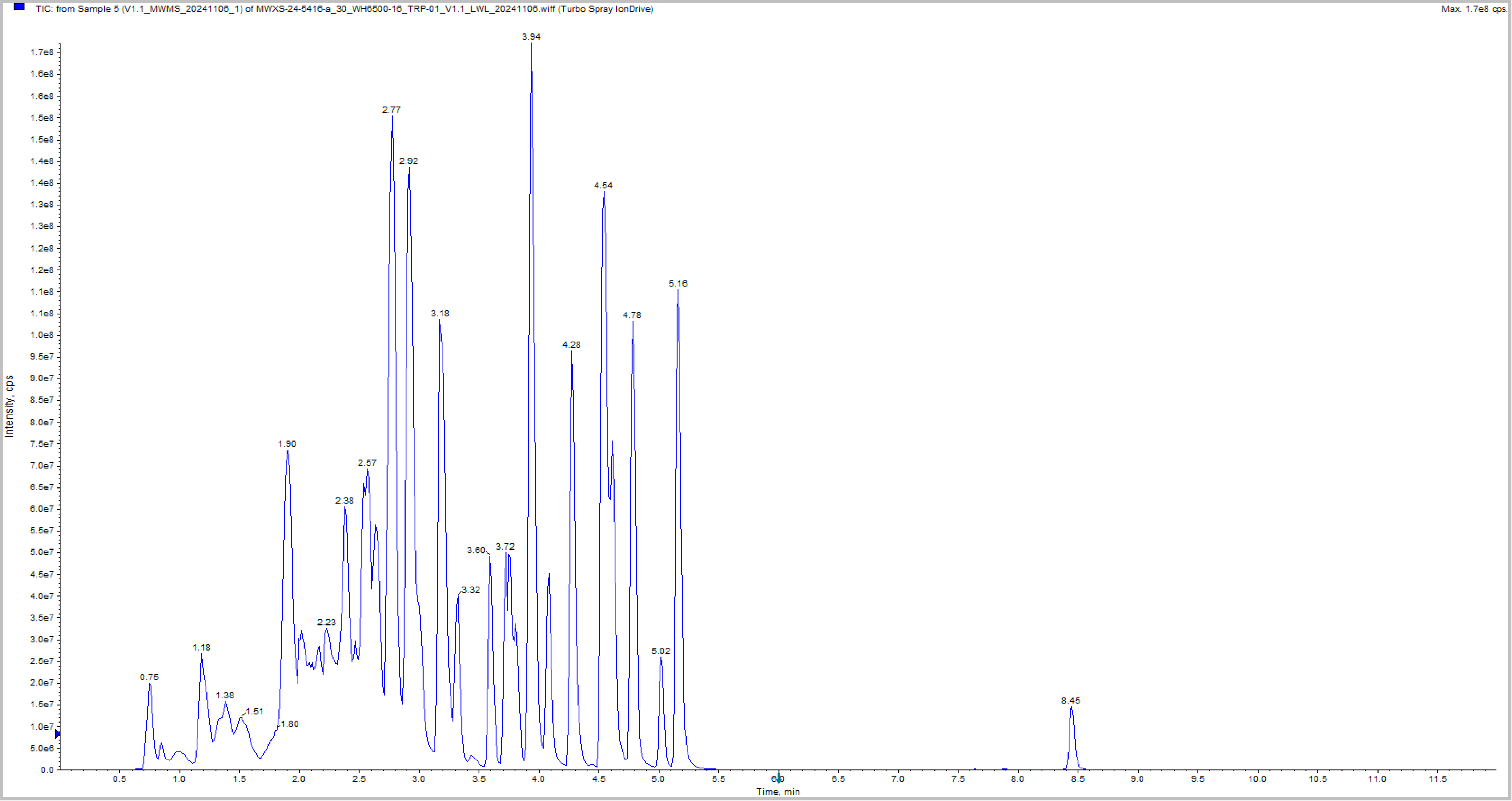

Supplement: Supplementary file 1 [file foods-14-03354-s001.zip › Supplementary Files/Supplementary Figure S3.tif]

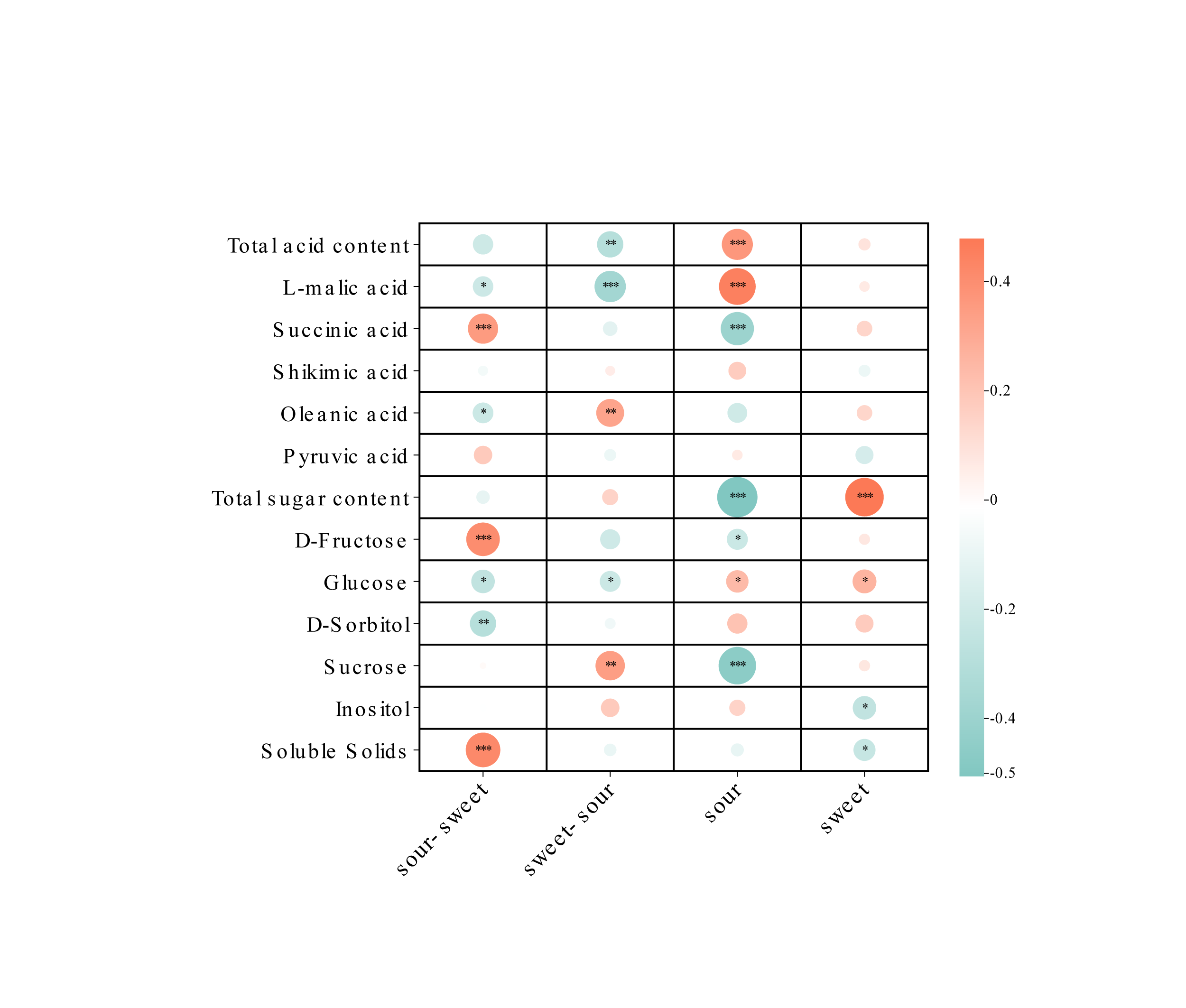

Supplement: Supplementary file 1 [file foods-14-03354-s001.zip › Supplementary Files/Supplementary Figure S4.tif]
